# Inhibiting Liver Autophagy and Promoting Hepatocyte Apoptosis by *Schistosoma japonicum* Infection

**DOI:** 10.3390/tropicalmed9020042

**Published:** 2024-02-04

**Authors:** Zhihao Yu, Tingting Jiang, Fangfang Xu, Jing Zhang, Yuan Hu, Jianping Cao

**Affiliations:** 1 National Institute of Parasitic Diseases, Chinese Center for Disease Control and Prevention, Chinese Center for Tropical Diseases Research, Shanghai 200025, China; yuzhihao0408@163.com (Z.Y.); jiangtingt16@163.com (T.J.); xufangfangx@163.com (F.X.); zhangjing@nipd.chinacdc.cn (J.Z.); 2 National Key Laboratory of Intelligent Tracking and Forecasting for Infectious Diseases, Key Laboratory on Parasite and Vector Biology, National Health Commission of the People’s Republic of China, Shanghai 200025, China; 3 World Health Organization Center for Tropical Diseases, Shanghai 200025, China; 4 The School of Global Health, Chinese Center for Tropical Diseases Research, Shanghai Jiao Tong University School of Medicine, Shanghai 200025, China

**Keywords:** *Schistosoma japonicum*, hepatocyte, autophagy, apoptosis, liver fibrosis

## Abstract

We established a mouse model of *Schistosoma japonicum* infection in order to study the effects of the infection on hepatocyte autophagy and apoptosis. We also stimulated HepG2 cells with soluble egg antigens (SEA) in vitro. At two, four, and six weeks post-infection, quantitative real-time PCR and Western blot (WB) were used to detect liver expression levels of autophagy and apoptosis-related proteins. HepG2 cells were treated with different concentrations of SEA. The changes in the levels of autophagy-related proteins and HepG2 cell apoptosis were detected. The *Lc3b*, *Beclin1*, *Atg7*, and *Atg12* mRNA levels were significantly lower at four and six weeks after infection than those in the uninfected group. At four and six weeks following infection, the levels of Beclin1, LC3BII/I, Atg7, and p62 proteins were considerably lower than those in the uninfected group. The protein levels of pro-apoptotic Bax and cleaved caspase 3 and fibrosis-related proteins α-SMA and collagen 3 in the liver post-infection were significantly higher than those in uninfected mice. HepG2 cells stimulated with SEA showed decreased levels of Beclin1, p62, and Atg7 proteins and significantly increased apoptosis rates. The findings demonstrated that following infection with *S. japonicum*, mice’s liver fibrosis worsened, hepatic autophagy was suppressed, and hepatocyte apoptosis was encouraged.

## 1. Introduction

Schistosomiasis is a neglected tropical disease that is endemic in 78 countries, infecting more than 200 million people worldwide and causing severe threats to human health [1]. The primary disease-causing pathogens in humans are *Schistosoma japonicum*, *S. mansoni,* and *S. haematobium*. In China, the predominant epidemic pathogen is *S. japonicum*. The prevalence of schistosomiasis has been effectively controlled in China. However, there are still many patients with advanced-stage schistosomiasis, which numbered 28,565 in 2022 [2]. Praziquantel is currently a widely used anti-schistosomal drug; however, there is a risk of drug resistance [3]. Following praziquantel treatment, patients with chronic schistosomiasis may still develop liver fibrosis [4]. After an *S. japonicum* infection, many eggs produced by the female worms are deposited in the liver and intestines. Soluble egg antigen (SEA) released from the eggs induces severe inflammatory reactions and the formation of egg granulomas, leading to aggravation and the development of liver fibrosis [5]. Determining the mechanisms underlying fibrosis generated by schistosomiasis is therefore crucial.

Physiological autophagy is a controlled process by which cells purge damaged organelles and aberrant cell components. Autophagy is crucial to maintaining cell homeostasis, while excessive and insufficient autophagy can induce various diseases [6,7]. There is evidence that autophagy is involved in liver fibrosis [8,9]. Autophagy dysfunction in hepatocytes correlated with the promotion of liver fibrosis, while restoring the expected level of autophagy in hepatocytes alleviated liver fibrosis [10]. Cell apoptosis is a type of controlled programmed cell death [11]. After activation of the pro-apoptotic signal, the genome is decomposed, and the intracellular proteins are lysed in the cell under the regulation of various cysteinases [12]. Autophagy can regulate the process of apoptosis by regulating the expression of apoptotic molecules or directly combining with apoptotic molecules [13,14]. In the liver, autophagy activity regulates hepatocyte apoptosis [15]. The degree of hepatocyte apoptosis and the course of liver disease are determined by the intricate interactions between hepatic autophagy and hepatocyte apoptosis [16].

Liver fibrosis develops by a multifaceted mechanism. The extracellular matrix produced by activated hepatic stellate cells serves as the main source of myofibroblasts [17]. The activation of hepatic stellate cells is regulated by a variety of cells in the liver [18,19,20]. Hepatocytes are the main parenchymal cells of the liver, accounting for about 80%. Hepatocyte proliferation is essential for liver homeostasis and regeneration [21]. Recent studies have shown that dead hepatocytes can release damage-associated molecular patterns (DAMPs) and secrete inflammatory factors to activate hepatic stellate cells and promote liver inflammation and the formation of liver fibrosis [22,23,24]. In the liver fibrosis model induced by carbon tetrachloride (CCL4), vitamin D receptors can indirectly inhibit the activation of hepatic stellate cells by enhancing hepatocyte autophagy, reducing hepatocyte damage, and alleviating liver fibrosis [25]. Studies showed that the deficiency of farnesoid X receptor in hepatocytes could disrupt bile acid homeostasis and inhibit autophagy, promote the production of liver inflammatory factors, and aggravate liver fibrosis in mice infected with *S. japonicum* [26]. Uncertainty exists on how *S. japonicum* infection affects apoptosis and liver autophagy.

A mouse model of *S. japonicum* infection was created for this investigation. Quantitative real-time reverse transcription PCR (qRT-PCR) and Western blotting were used to detect the changes in liver autophagy, apoptosis, and liver fibrosis protein levels at two, four, and six weeks after infection. HepG2 cells were stimulated with different concentrations of SEA in vitro, and the levels of autophagy-related proteins and the rates of hepatocyte apoptosis were detected. Our results will offer an experimental foundation for future research on the regulatory function of hepatocyte autophagy in *S. japonicum*-induced liver fibrosis.

## 2. Materials and Methods

### 2.1. Ethics Statement

All animal experiments were carried out according to the protocol recommended by the Laboratory of Animal Welfare and Ethics Committee (LAWEC) of China. The LAWEC of the National Institute of Parasitic Diseases, Chinese Centre for Disease Control and Prevention, approved the animal experimental protocol (approval ID: IPD-2020-10).

### 2.2. Animals and Parasites

Female C57BL/6 mice were purchased from Shanghai Lingchang Biotechnology Co., Ltd. (Shanghai, China). They were 6–8 weeks old and had a body weight of 20 ± 2 g. The mice were housed in an SPF-grade animal room. The *S. japonicum* cercaria was provided by Anhui Institute of Schistosomiasis Control. Mice were percutaneously infected with cercaria after shaving the skin of the abdomen. The HepG2 (human hepatoma) cell line was purchased from Ubigene Biosciences (Guangzhou, China).

### 2.3. Reagents

The bicinchoninic acid (BCA) protein quantification assay kit was purchased from Beijing Solarbio Science & Technology Co., Ltd. (Beijing, China). The Annexin V-fluorescein isothiocyanate (FITC)/propidium iodide (PI) cell apoptosis detection kit was purchased from Absin Bioscience Inc. (Shanghai, China). Trizol was purchased from Takara Bio Inc. (Beijing, China). Hyper ScriptTMIII RT SuperMix and Universal SYBR qPCR Mix were provided by Enzy Artisan (Shanghai, China). Rapamycin and chloroquine were purchased from MedChemExpress (Monmouth Junction, NJ, USA). Rabbit anti-microtubule-associated proteins 1A/1B light chain 3B (LC3B) (3868S), p62 (5114S), glyceraldehyde-3-phosphate dehydrogenase (GAPDH) (5174S), Beclin1 (3495S), cleaved caspase-3 (9661S), and alpha-smooth muscle actin (α-SMA) (19245S) antibodies were purchased from Cell Signaling Technology (CST, Danvers, MA, USA). Rabbit anti-autophagy-related 7 (Atg7) (ab52472) antibodies were purchased from Abcam (Cambridge, MA, USA). Rabbit anti-BCL2 associated X protein (Bax) (50599-2-Ig) and mouse anti-β-actin (81115-1-RR) antibodies were purchased from ProteinTech (Wuhan, China). Rabbit anti-collagen III a1 antibodies were purchased from Bioss (Beijing, China).

### 2.4. SEA Preparation

A small amount of *S. japonicum* eggs were added to sterile PBS, then placed in a −80 °C refrigerator overnight, and repeatedly frozen and thawed 3–5 times. SEA was homogenized in an ice bath and then crushed ultrasonically for 20 min at 4 °C. The homogenate of eggs was centrifuged at 10,000× *g* for 30 min, and the supernatant was collected. The supernatant was filtered to remove bacteria and separate packing. The protein concentration was detected using the BCA method for subsequent use.

### 2.5. Establishment of the S. japonicum-Infected Mouse Model

Forty-eight C57BL/6 mice were divided into infected and uninfected groups (twenty-four mice per group). Mice were anesthetized via an intraperitoneal injection of 1% sodium pentobarbital. After shaving off their abdominal skin, the mice were infected with 20 ± 1 cercaria percutaneously. Eight mice from each group were anesthetized and then dissected at two, four, and six weeks post-infection, respectively.

### 2.6. In Vitro Stimulation of HepG2 Cells with SEA

HepG2 cells were cultured in DMEM medium containing 10% FBS under 5% CO_2_ at 37 °C. The cells were passaged at a ratio of 1:2 when they reached 80–90% confluency after digestion with 0.25% trypsin. The SEA was added into the HepG2 cell culture system at final concentrations of 80, 160, and 320 μg/mL, respectively. After stimulation for 24 and 48 h, the levels of autophagy-related proteins and the apoptosis rates of HepG2 cells were detected.

### 2.7. The Expression of Autophagy-Related Genes

Liver tissues from mice infected with *S. japonicum* for two, four, and six weeks were ground in liquid nitrogen and fully lysed using Trizol. After adding chloroform, the samples were inverted and mixed and then centrifuged at 12,000× *g* for 15 min. Subsequently, the upper solution was retained, mixed with isopropanol, and left at room temperature for 10 min. After centrifugation at 12,000× *g* for 10 min, the supernatant was discarded, and the precipitate was washed with 75% ethanol. After centrifugation, the precipitate RNA dissolved in DEPC water. The mRNA concentration was measured with a NanoDrop2000 from Thermo Fisher Scientific (Waltham, MA, USA) spectrophotometer, and mRNA OD260/280 was considered to be pure when it was between 1.8 and 2.05. To remove genomic DNA, total RNA 1 μg, 8 × gDNAse 2 μL, and RNase-free ddH_2_O supplementation 16 μL were mixed at 42 °C for 2 min. To obtain cDNA, 4 μL of 5 × RT super mix (Takara, Shiga, Japan) was added to 16 μL of RNA production after removing genomic DNA and then incubated at 37 °C for 15 min and at 85 °C for 5 s.

The qPCR step was used to determine the mRNA levels of the target genes in liver tissues using Fast SYBR Green master Mix (Bio-Rad, Hercules, CA, USA). The qPCR reaction system included 2 × S6 Universal SYBR qPCR Mix (10 μL), upstream primers (1 μL), downstream primers (1 μL), the prepared cDNA (2 μL), and ddH_2_O (6 μL). The primers were synthesized by Enzy Artisan Co., Ltd (Shanghai, China). and are listed in Table 1. The qPCR reaction conditions were as follows: 38 cycles of 95 °C for 30 s, 95 °C for 5 s, and 60 °C for 30 s. After the cycle threshold (Ct) value was obtained, the relative expression of the target gene was calculated using the 2^−ΔΔCt^ method [27].

### 2.8. The Expression of Autophagy, Apoptosis, and Fibrosis-Related Proteins

HepG2 cells were stimulated with high concentrations of SEA (80, 160, and 320 μg/mL) for 24 and 48 h. After adding a protease inhibitor cocktail (Beyotime Biotechnology, Shanghai, China), HepG2 cells or liver tissues were lysed using lysis buffer (Shanghai Epizyme Biomedical Technology Co., Ltd., Shanghai, China) for 30 min on ice. The lysates were centrifuged, and the supernatants were used for protein quantification through the BCA method. After adding the sample buffer, the samples were boiled in a metal bath at 100 °C for 5 min. The proteins in the samples were separated using 12% sodium dodecyl sulfate–polyacrylamide gel electrophoresis at 90 V for 90 min. Then, the separated proteins were transferred to polyvinylidene fluoride (PVDF) membranes, blocked at room temperature for 1 h, and added with rabbit primary antibodies (1:1000). A horseradish peroxidase-conjugated anti-mouse IgG antibody (7074S, CST; 7076S, CST) was used as the secondary antibody. The immunoreactive protein was detected using an ECL chemiluminescence solution ordered from Epizyme Biomedical Technology Co., Ltd (Shanghai, China). The protein bands were semiquantitatively analyzed using Image J 1.53t software (NIH, Bethesda, MD, USA). The amount of sample in each channel was about 30 μg, and at least three biological replicates were performed for each protein.

### 2.9. Determining Apoptosis Rates of HepG2 Cells

HepG2 cells were stimulated with SEA (the final concentrations were 80, 160, and 320 μg/mL) for 48 h. After being digested with 0.25% trypsin, the cells were washed twice and centrifuged at 200× *g* for 5 min at room temperature. The supernatant was discarded. The precipitated cells were resuspended in 200 μL of binding buffer, mixed with 5 μL of Annexin V-FITC, incubated at room temperature for 15 min in the dark, and then mixed with 5 μL of PI. After resuspension, cells were detected using a FACS Verse flow cytometer from BD Biosciences (Franklin Lake, NJ, USA). The experimental results were analyzed using Flow-Jo10.8.1 software (TreeStar Inc., Ashland, OR, USA).

### 2.10. Statistical Analysis

Data analysis was performed using GraphPad Prism Version 10.0.2 (GraphPad Inc., La Jolla, CA, USA). Differences were analyzed using a non-parametric one-way analysis of variance. Data are presented as the mean ± standard deviation. *p* < 0.05 indicates a significant difference.

## 3. Results

### 3.1. The Level of Liver Autophagy Decreased Significantly in Mice Infected with S. japonicum

**Figure 1:**
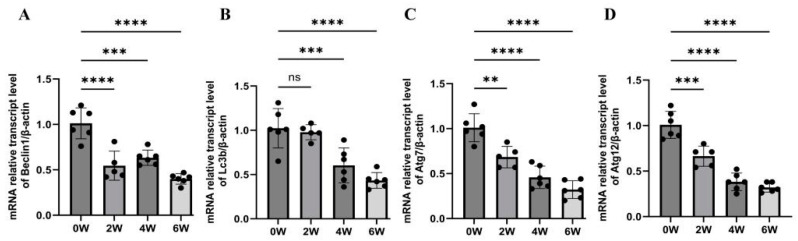
Changes in liver autophagy-related genes in mice infected with *Schistosoma japonicum*. After two, four, and six weeks of infection, mouse livers were collected for follow-up experiments. (**A**) qPCR results of Beclin1 expression in the liver after infection. (**B**) qPCR results of Lc3b expression in the liver after infection. (**C**) qPCR results of Atg7 expression in the liver after infection. (**D**) qPCR results of Atg12 expression in the liver after infection. ** *p* < 0.01; *** *p* < 0.001; **** *p* < 0.0001; ns, no significant.

The qPCR results showed that the Lc3b mRNA level in the liver tissue of mice infected with *S. japonica* was 0.98 ± 0.085 at two weeks, which was not significantly different from that of the uninfected group (1.00 ± 0.22) (*p* > 0.05). The *Lc3b* mRNA expression in mouse liver tissue was 0.60 ± 0.20 and 0.43 ± 0.089 at four and six weeks after infection, respectively, which was significantly lower than that before infection (1.0 ± 0.22) (*F* = 17.85, *p* < 0.01). The mRNA expression levels of *Beclin1* were 0.55 ± 0.16, 0.63 ± 0.084, and 0.40 ± 0.058 at two, four, and six weeks after infection, respectively. These were significantly lower than those before infection (1.0 ± 0.17) (*F* = 25.85, *p* < 0.01). The mRNA expression levels of Atg7 were 0.68 ± 0.12, 0.46 ± 0.12, and 0.32 ± 0.099, respectively, which were significantly lower than those before infection (1.0 ± 0.16) (*F* = 33.79, *p* < 0.01). The mRNA expression levels of Atg12 were 0.66 ± 0.11, 0.38 ± 0.096, and 0.32 ± 0.051, respectively, which were significantly lower than those before infection (1.0 ± 0.15) (*F* = 51.55, *p* < 0.01) (Figure 1). These results show that the expression of autophagy-related genes decreased significantly at two, four, and six weeks after *S. japonicum* infection.

**Figure 2:**
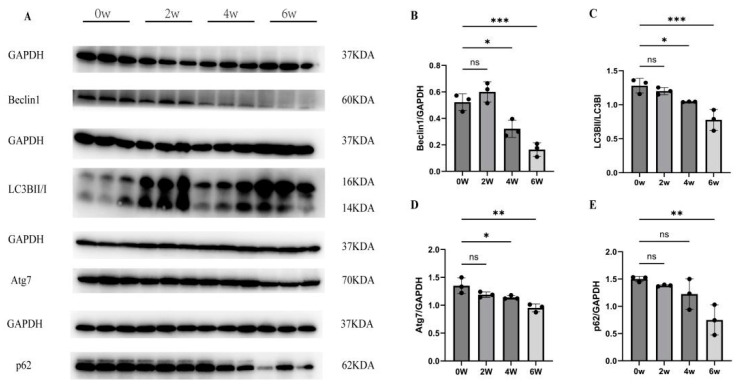
Changes in liver autophagy protein levels in mice infected with *S. japonicum*. (**A**–**E**) Western blot results of autophagy of Beclin1, LC3BII/I, Atg7, and p62 in the liver after two, four, and six weeks of infection. * *p* < 0.05; ** *p* < 0.01; *** *p* < 0.001; ns, no significant.

The protein expression levels of LC3BII/I at four and six weeks after infection were 1.0 ± 0.0039 and 0.78 ± 0.15, which were significantly lower than those before infection (1.3 ± 0.026) (*F* = 20.03, *p* < 0.01). The protein expression levels of Beclin1 were 0.32 ± 0.066 and 0.16 ± 0.05, significantly lower than (0.52 ± 0.026) before infection (*F* = 27.74, *p* < 0.01). The protein expression levels of Atg 7 were 1.1 ± 0.034 and 0.95 ± 0.072, significantly lower than those before infection (1.4 ± 0.14) (*F* = 11.37, *p* < 0.01). The protein expression level of p62 was 0.75 ± 0.28 six weeks after infection, lower than that before infection (1.5 ± 0.05) (*F* = 8.23, *p* < 0.01) (Figure 2). The results show that the expression of autophagy-related proteins decreased significantly at four and six weeks after *S. japonicum* infection.

### 3.2. The Levels of Liver Apoptosis and Liver Fibrosis Increased Significantly in S. japonicum-Infected Mice 

At four and six weeks post-infection, the mRNA levels of Bax were 1.40 ± 0.05 and 1.50 ± 0.06, which were significantly higher than those of the uninfected group (1.0 ± 0.02) (*F* = 40.59, *p* < 0.01). The mRNA levels of collagen I were 5.1 ± 0.76 and 7.8 ± 3.6, which were significantly higher than those of the uninfected group (1.20 ± 0.62) (*F* = 13.8, *p* < 0.01). The mRNA levels of collagen III were 5.4 ± 3.0 and 36 ± 4.1, which were significantly higher than those of the uninfected group (1.0 ± 0.34) (*F* = 192.3, *p* < 0.01). The mRNA levels of α-SMA were 4.1 ± 0.82 and 13.0 ± 3.7, which were significantly higher than those of the uninfected group (1.0 ± 0.36) (*F* = 34.43, *p* < 0.01). 

The Western blot results showed that the protein expression levels of cleaved caspase 3 were 1.3 ± 0.099 and 1.0 ± 0.22 at four and six weeks after infection, which were significantly higher than those of the uninfected group (0.12 ± 0.083) (*F* = 54.01, *p* < 0.01). At two, four, and six weeks after infection, Bax expressions were 0.58 ± 0.037, 0.56 ± 0.044, and 0.62 ± 0.05, significantly higher than those of the uninfected group (0.39 ± 0.11) (*F* = 6.77, *p* < 0.05). The results suggest that the level of liver apoptosis significantly increased at two, four, and six weeks after *S. japonicum* infection.

**Figure 3:**
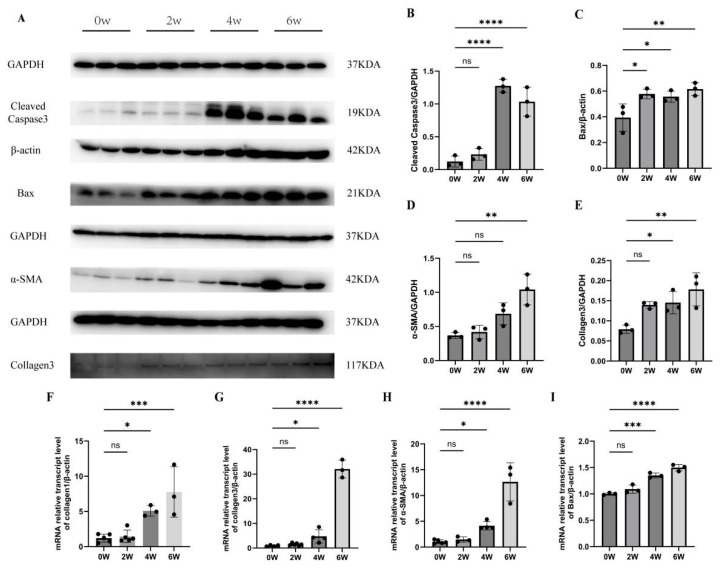
Changes in liver apoptosis and fibrosis levels in mice infected with *S. japonicum*. (**A**–**C**) Cleaved caspase 3 and Bax expressions at two, four, and six weeks post-infection in the liver. (**A**,**D**,**E**) α-SMA and collagen 3 protein expressions at two, four, and six weeks post-infection. (**F**–**I**) Collagen I, collagen III, α-SMA, and Bax mRNA expressions at two, four, and six weeks post-infection. * *p* < 0.05; ** *p* < 0.01; *** *p* < 0.001; **** *p* < 0.0001; ns, no significant.

The protein expressions of collagen 3 were 0.15 ± 0.028 and 0.18 ± 0.041 at four and six weeks post-infection, which were significantly higher than those of the uninfected group (0.079 ± 0.010) (*F* = 7.721, *p* < 0.01). At six weeks post-infection, α-SMA expression was 1.0 ± 0.23, significantly higher than that of the uninfected group (0.37 ± 0.043) (*F* = 12.99, *p* < 0.01). The results indicate that the degree of liver fibrosis increased significantly at four and six weeks after infection, consistent with the changing trend of liver apoptosis (Figure 3).

### 3.3. The Autophagy Level of HepG2 Cells Decreased after SEA Stimulation

**Figure 4:**
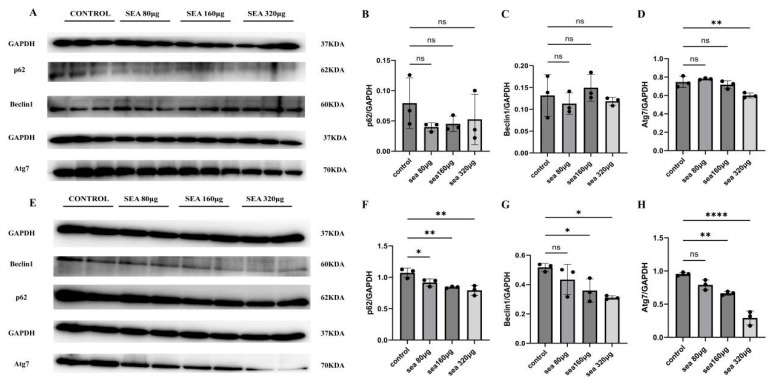
The expressions of autophagy-related proteins in HepG2 cells after stimulation with SEA of different concentrations. (**A**–**D**) The expressions of p62, Beclin1, and Atg7 after being stimulated for 24 h with SEA in HepG2. (**E**–**H**) The expressions of p62, Beclin1, and Atg7 after being stimulated for 48 h with SEA in HepG2 * *p* < 0.05; ** *p* < 0.01; **** *p* < 0.0001; ns, no significant.

After being treated with SEA (80, 160, and 320 μg/mL) for 24 h, the expressions of Beclin1 and p62 protein in HepG2 cells were not significantly different from those in the control group (*p* > 0.05). When the concentration of SEA was 320 μg/mL, Atg7 protein was 0.60 ± 0.028, significantly lower than that of the control group (0.75 ± 0.060) (*F* = 11.58, *p* < 0.01). After being stimulated for 48 h with SEA (80, 160, and 320 μg/mL), p62 protein levels were 0.92 ± 0.058, 0.84 ± 0.013, 0.79 ± 0.078, significantly lower than those of the control group (1.1 ± 0.076) (*F* = 11.51, *p* < 0.01). After being stimulated with SEA of 160 μg/mL and 320 μg/mL for 48 h, Beclin1 expressions were 0.36 ± 0.080 and 0.31 ± 0.013, significantly lower than those of the control group (0.52 ± 0.027) (*F* = 5.451, *p* < 0.01). The expression of Atg7 protein was 0.66 ± 0.029 and 0.29 ± 0.11, respectively, which was significantly lower than that of the control group (0.95± 0.026) (*F* = 52.00, *p* < 0.01) (Figure 4). The results suggest that SEA can decrease the expression of autophagy-related proteins in HepG2 cells.

### 3.4. Apoptosis Increased after Being Stimulated with SEA in HepG2 Cells

**Figure 5:**
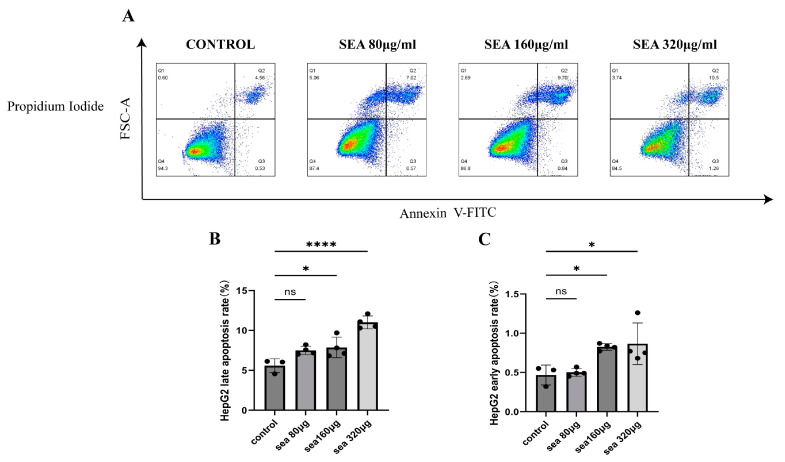
The apoptosis rates of HepG2 cells after being stimulated with SEA from 80 to 320 μg/mL for 48 h. (**A**–**C**) The early and late apoptotic rates after being stimulated with 80, 160, and 320 μg/mL SEA. * *p* < 0.05; **** *p* < 0.0001; ns, no significant. Red indicates high cell density, green indicates medium cell density, and blue indicates low cell density.

After being stimulated with SEA (80 µg/mL) for 48 h, the early and late apoptosis rates of HepG2 cells were 0.5 ± 0.05% and 7.5 ± 0.53%, which were not significantly different from those without stimulation (0.47 ± 0.13% and 5.6 ± 0.88%, *p* > 0.05). After being stimulated with SEA (160, 320 μg/mL) for 48 h, the early apoptosis rates were 0.83 ± 0.042% and 0.87 ± 0.27%, significantly higher than those without stimulation (0.47 ± 0.13%, *p* < 0.05). At the concentrations of 160 and 320 μg/mL SEA, the late apoptosis rates of HepG2 cells were 7.9 ± 1.3% and 11.0 ± 0.81%, significantly higher than those without stimulation (5.6 ± 0.88) % (*p* < 0.05) (Figure 5). This suggests that SEA stimulation can promote the early and late apoptosis of HepG2 cells in a dose-dependent manner.

## 4. Discussion

Schistosomiasis japonica is a zoonotic parasite illness that is primarily seen in China, the Philippines, Indonesia, and other countries [28]. It has a significant negative impact on society and puts human health in grave peril. When the host is exposed to water containing cercariae, the cercariae can penetrate the skin directly into the host body and then migrate to the host’s portal system to develop into adult worms. After the male and female worms embrace each other, the female worm lays eggs, and the eggs secrete a large amount of SEA, inducing a robust immune response [29]. Under the continuous influence of SEA, the antigen and antibody produced by the host form a complex around the egg, causing local allergic reactions and granuloma formation. The extracellular matrix then over-deposits and progresses to cause liver fibrosis, which is accompanied by hepatosplenomegaly, portal hypertension, and other symptoms [5,30].

In cells under stress, autophagy is a highly conserved catabolic process that occurs under different circumstances. The activity of autophagy is low under physiological conditions but significantly increased under cell starvation, hypoxia, and other stress conditions [31]. Autophagy mainly has a cytoprotective function and plays an essential role in reducing apoptosis, oxidative stress, endoplasmic reticulum stress, and inflammation [31,32,33]. In recent years, the protective role of autophagy has been demonstrated in non-alcoholic fatty liver disease (NAFLD), liver fibrosis, and other liver diseases [34,35,36]. In the CCL4-induced liver fibrosis model, oroxylin A significantly upregulated autophagy expression and alleviated liver fibrosis.

In contrast, the anti-fibrosis activity of oroxylin A was eliminated when autophagy was inhibited with the particular inhibitor 3-methyladenine (3-MA) [37]. In the liver, about 80% of the cells are hepatocytes. Autophagy can reduce hepatocyte damage, and inhibition of autophagy can promote liver injury and inflammation [38]. Research has shown that hepatocyte autophagy can alleviate the process of liver fibrosis by attenuating epithelial–mesenchymal transition [35] and inhibiting cell necrosis and apoptosis [39].

Cells use autophagy as a vital defense mechanism against damage. Hepatocytes with less autophagy will have increased oxidative stress and inflammatory response, which will lead to more cell death [35,38,40]. This study showed that liver autophagy-related Beclin1, p62, LC3BII/I, Atg7, and Atg12 levels decreased significantly at two, four, and six weeks post-infection. Schistosoma migrates to the liver at two weeks post-infection, and female worms lay eggs in the portal system at four weeks post-infection. A large number of eggs are deposited in the liver. Therefore, the decrease in liver autophagy is related to liver injury induced by the schistosomula and eggs. Liver fibrosis-related indexes of α-SMA, collagen 1, and collagen 3 were significantly increased at four and six weeks post-infection. Egg production began at four weeks and reached a peak at six weeks post-infection. It was suggested that liver fibrosis was related to egg deposition in the liver. In in vitro experiments, the expression levels of autophagy-related proteins (Beclin1, Atg7, and p62) decreased significantly after stimulation with SEA (>80 μg/mL). It was consistent with the results of infected mice. According to conjecture, schistosome infection can cause significant inflammation, impair liver homeostasis, decrease hepatocyte autophagy, and encourage liver fibrosis.

Bax is a pro-apoptotic protein that can mediate the release of Cyt-c through the mitochondrial pathway to activate caspase 3. Cleaved caspase 3 is the activated form of caspase 3. The expressions of Bax and cleaved caspase 3 increasing indicates that apoptosis increased [41]. In this investigation, four and six weeks after infection, the liver’s levels of the pro-apoptotic proteins Bax and cleaved caspase 3 dramatically increased. Moreover, the changes in Bax protein occurred earlier than in cleaved caspase 3. When HepG2 cells were stimulated with SEA of 80 μg/mL, early apoptosis and late apoptosis (necrosis) levels had no change compared to those before stimulation. When HepG2 cells were stimulated by SEA of 160 and 320 μg/mL, early apoptosis levels and necrosis levels significantly increased in a dose-dependent manner. It could be speculated that the proportion of apoptosis and necrosis of hepatocytes that increased was induced by SEA stimulation. It can be the primary cause of the escalation of liver fibrosis and inflammation.

Hepatocyte autophagy has been shown in recent years to be able to reduce the amount of necrosis and apoptosis. Rapamycin, an autophagy activator, inhibits hepatocyte apoptosis by enhancing emodin-induced hepatocyte autophagy. The apoptosis rate increased significantly after treatment with the autophagy inhibitor 3-MA [42]. In NAFLD, increasing hepatocyte autophagy can reduce endoplasmic reticulum stress, decrease apoptosis levels, and improve liver injury and inflammation [36]. In CCL4-induced liver fibrosis, small p97/vcp-interacting protein (SVIP) activated autophagy and alleviated liver fibrosis. After SVIP is depleted by siRNA, hepatocytes are more sensitive to CCL4 toxicity [33].

Many studies on the mechanism of schistosomiasis-induced liver fibrosis have concentrated on the control of hepatic stellate cells activated by immune cells, such as NK cells [43], macrophages [44], eosinophils [45], and γδT cells [46]. However, less attention has been paid to hepatocyte-regulating hepatic stellate cell activation. In this study, liver autophagy and apoptosis changes were studied by establishing infected mice and HepG2 cells stimulated with SEA. Our results indicated that schistosomiasis infection could inhibit liver autophagy, promote apoptosis or necrosis of liver cells, and aggravate liver fibrosis. The limitation of this study is that autophagy inhibitors and activators were not directly used in schistosomiasis infection models to verify whether regulating autophagy in schistosomiasis infection models can improve fibrosis. However, it is anticipated that controlling hepatocyte autophagy will eventually be used as a therapeutic approach to treat liver fibrosis brought on by *S. japonicum* infection.

## Figures and Tables

**Table 1:** Primer sequences for qPCR.

ID Number	Gene Name	Primer Sequence (5′→3′)
11461	Actb (β-actin)	F: CATTGCTGACAGGATGCAGAAGG
		R: TGCTGGAAGGTGGACAGTGAGG
67443	Lc3b	F: GTCCTGGACAAGACCAAGTTCC
		R: GAGGAAGAAGGCTTGGTTAGCA
56208	Beclin1	F: GGAGGGGTCTAAGGCGTCCAG
		R: TCTTGAAGCTCGTGTCCAGTTTCAG
74244	Atg7	F: CCTGTGAGCTTGGATCAAAGGC
		R: GAGCAAGGAGACCAGAACAGTG
67526	Atg12	F: GCTGAAGGCTGTAGGAGACACTC
		R: AGTCAATGAGTCCTTGGATGGTC
12028	Bax	F: AGGATGCGTCCACCAAGAAGCT
		R: TCCGTGTCCACGTCAGCAATCA
11475	α-Sma	F: CTGGTATTGTGCTGGACTCTG
		R: GATCTTCATGAGGTAGTCGGTG
12842	Collagen-I	F: CCTCAGGGTATTGCTGGACAAC
		R: TTGATCCAGAAGGACCTTGTTTG
12825	Collagen-III	F: GACCAAAAGGTGATGCTGGACAG
		R: CAAGACCTCGTGCTCCAGTTAG

## Data Availability

Data are contained within the article.
