# Correction: Yu et al. Inhibiting Liver Autophagy and Promoting Hepatocyte Apoptosis by *Schistosoma japonicum* Infection. *Trop. Med. Infect. Dis.* 2024, *9*, 42

**DOI:** 10.3390/tropicalmed9030062

**Published:** 2024-03-18

**Authors:** Zhihao Yu, Tingting Jiang, Fangfang Xu, Jing Zhang, Yuan Hu, Jianping Cao

**Affiliations:** 1 National Institute of Parasitic Diseases, Chinese Center for Disease Control and Prevention, Chinese Center for Tropical Diseases Research, Shanghai 200025, China; yuzhihao0408@163.com (Z.Y.); jiangtingt16@163.com (T.J.); xufangfangx@163.com (F.X.); zhangjing@nipd.chinacdc.cn (J.Z.); 2 National Key Laboratory of Intelligent Tracking and Forecasting for Infectious Diseases, Key Laboratory on Parasite and Vector Biology, National Health Commission of the People’s Republic of China, Shanghai 200025, China; 3 World Health Organization Center for Tropical Diseases, Shanghai 200025, China; 4 The School of Global Health, Chinese Center for Tropical Diseases Research, Shanghai Jiao Tong University School of Medicine, Shanghai 200025, China

In the published publication [1], there was an error regarding the affiliations for the authors. The updated affiliations should be as follows:

Zhihao Yu ^1,2,3^, Tingting Jiang ^1,2,3^, Fangfang Xu ^1,2,3^, Jing Zhang ^1,2,3^, Yuan Hu ^1,2,3,^* and Jianping Cao ^1,2,3,4,^*

^1^  National Institute of Parasitic Diseases, Chinese Center for Disease Control and Prevention, Chinese Center for Tropical Diseases Research, Shanghai 200025, China; yuzhihao0408@163.com (Z.Y.); jiangtingt16@163.com (T.J.); xufangfangx@163.com (F.X.); zhangjing@nipd.chinacdc.cn (J.Z.)
^2^  National Key Laboratory of Intelligent Tracking and Forecasting for Infectious Diseases, Key Laboratory on Parasite and Vector Biology, National Health Commission of the People’s Republic of China, Shanghai 200025, China
^3^  World Health Organization Center for Tropical Diseases, Shanghai 200025, China
^4^  The School of Global Health, Chinese Center for Tropical Diseases Research, Shanghai Jiao Tong University School of Medicine, Shanghai 200025, China

*  Correspondence: huyuan@nipd.chinacdc.cn (Y.H.); caojp@chinacdc.cn (J.C.)

‘*Schistosoma japonicum*’ in the title should be italicized.

The authors state that the scientific conclusions are unaffected. This correction was approved by the Academic Editor. The original publication has also been updated.
